# Multisystem involvement in hydatid disease: a case report of disseminated echinococcosis

**DOI:** 10.3389/fpara.2025.1630827

**Published:** 2025-10-06

**Authors:** Hanamanthraya Mallannagouda, Shivanand Melkundi, Sanjana Devarmani, Vedashree V. Tiwari

**Affiliations:** Department of Radiodiagnosis, Mahadevappa Rampure Medical College, Kalaburagi, Karnataka, India

**Keywords:** hydatid disease, disseminated hydatid cyst, spine, intra-osseous, US, CECT, MRI

## Abstract

**Background:**

Hydatid disease is a zoonosis caused by the larval stage of *Echinococcus granulosus*, most often affecting the liver and lungs. Disseminated hydatidosis is rare, accounting for <10% of cases.

**Case presentation:**

We present a 28-year-old man with paraplegia and abdominal pain. He was first diagnosed with hydatid disease at a government hospital 3 years earlier and presented to us with only an ultrasound (US) report. No serology reports were furnished. He had deferred surgery due to financial constraints. At current presentation, US, computed tomography (CT), and magnetic resonance imaging (MRI) demonstrated multiple cysts across the pelvis, retroperitoneum, spine, mediastinum, neck, and extremities. Imaging morphology was consistent with the WHO-IWGE (Informal Working Group on Echinococcosis) CE1–CE3 hydatid cysts. Differentials including abscess, cysticercosis, necrotic metastases, and lymphangioma were ruled out based on the absence of contrast enhancement, calcification pattern, and clinical correlation.

**Treatment and outcome:**

Surgery was advised, but was declined. PAIR (puncture, aspiration, injection, re-aspiration) was contraindicated due to multivesicular bone and spinal cysts. The patient was managed with oral albendazole. Follow-up data are currently unavailable.

**Conclusion:**

This case highlights disseminated hydatid disease with an unusual spinal and soft tissue involvement. Multimodality imaging is pivotal for diagnosis and treatment planning. Awareness of the imaging features is essential for timely recognition and management.

## Introduction

The word “hydatid” in Greek translates to “a drop of water.” Hydatid disease is a zoonotic infection commonly seen in endemic countries such as India, caused by the tapeworm *Echinococcus granulosus*. Humans are an accidental intermediate host (Karami et al., 2022). Multiple studies have reported that the annual incidence of human cystic echinococcosis in India ranges from 1 to 200 per 100,000 population, with a higher prevalence in regions such as Kashmir, Andhra Pradesh, Tamil Nadu, and Central India (Singh et al., 2014).

Echinococcosis is transmitted through the fecal–oral route either by direct contact with animals or through the ingestion of parasitic eggs in contaminated food, water, or soil (Alshoabi et al., 2023). Hydatid cysts are most commonly seen in the liver (75%) and the lungs (15%) (Ito et al., 2017). Dissemination or the involvement of other organs by the hydatid disease is rare. However, when present, it can involve any organ in the body.

Imaging is essential for diagnosing and delineating the extension of the disease. Ultrasound (US) is the first-line modality, which rapidly identifies hydatid cysts through features such as daughter cysts, septations, and hydatid sand and is also useful for follow-up. Computed tomography (CT) complements US, and it is superior in detecting wall calcification and bony involvement and in revealing complications, particularly in areas poorly visualized by US or in patients with big body habitus. Magnetic resonance imaging (MRI) provides the best soft tissue contrast, clearly demonstrating cyst–neural element relationships, wall defects, and extradural or muscular extension. It is especially valuable for spinal and craniovertebral involvement (Mehta et al., 2016).

Hydatid cysts can have an extremely wide range of presentation, from purely cystic to solid, unilocular or multilocular, and with or without calcification. The findings also vary from organ to organ, disease stage, and the host response.

Based on the imaging findings, the WHO-IWGE (Informal Working Group on Echinococcosis) classified hydatid cysts into five types (Botezatu et al., 2018). In the classification of hydatid cysts, cystic echinococcosis CE1 and CE2 represent the active stages. CE1 is characterized by an anechoic uniloculated cyst with fine internal echoes, known as hydatid sand. CE2 presents as a cyst with multiple septations, where the septa represent daughter cysts (Pakala et al., 2016), creating the classic “honeycomb” or “rosette” sign. The transitional stage, CE3, is divided into two subtypes: CE3A features a unilocular cyst with detached laminated membranes, producing the “water lily” sign, while CE3B is defined by daughter cysts within a solid matrix. In the degenerative stage, CE4, the cyst contains mixed echogenic contents (both hypo- and hyperechoic), forming the “ball of wool” sign, and there are no daughter cysts present. Finally, CE5, the inactive stage, is marked by a cyst with a partially or totally calcified wall (Junghanss et al., 2008).

## Case presentation

A 28-year-old man presented to our hospital with progressive lower limb weakness since 3 months. He also reported colicky lower abdominal pain, which was insidious in onset, gradually worsened, and was associated with mild fever and constipation.

The patient had similar complaints of abdominal pain 2–3 years ago and visited a different government hospital for the same, where he underwent an US scan, which showed a multiloculated cystic lesion in the pelvic cavity and no other cystic lesions in the liver. The patient was diagnosed with extra-hepatic hydatid disease based on the imaging findings. He did not retain prior images or serology reports. The patient was advised surgical removal, but he refused due to financial constraints. He was instead started on oral albendazole (400 mg), but was inconsistent with the medication as well.

The present general physical examination recorded a blood pressure of 110/80, and a pulse rate of 78 bpm. His respiratory rate and oxygen saturation were normal.

Local examination of the abdomen revealed a tense abdomen with multiple palpable soft masses in the pelvic cavity of varying sizes. There were visible lumps in the supra-sternal notch extending inferiorly into the retrosternal space. Palpable masses were also noted in the anterior chest wall bilaterally inferior to the clavicles, the extensor aspects of the bilateral upper limbs, and the anterior aspect of the right proximal thigh. Routine blood investigations including complete blood count, renal function test, liver function test, electrolytes, and urine routine were normal.

**Figure 1:**
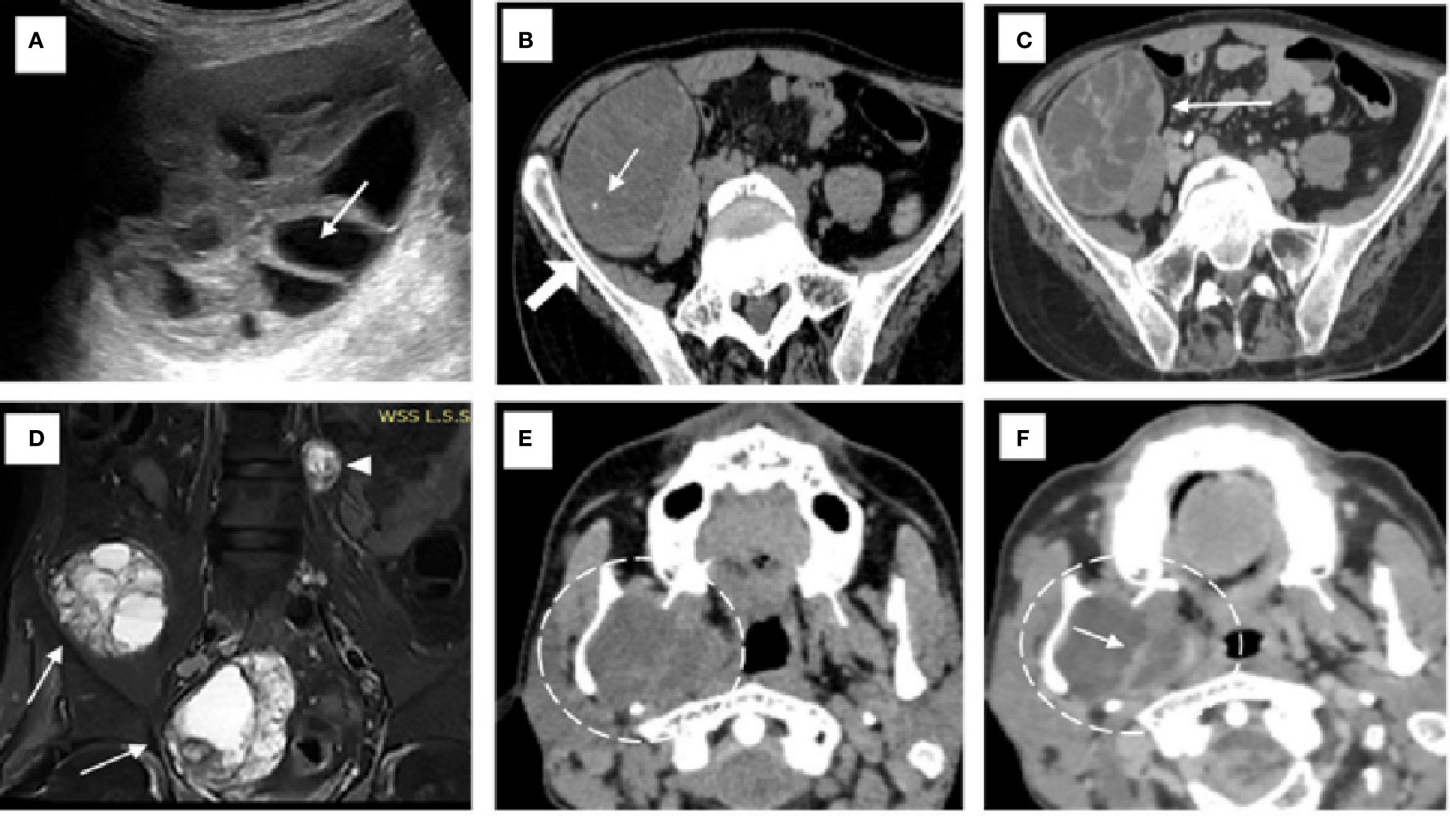
Imaging findings of hydatid disease involving the pelvis and mandible. **(A)** On ultrasound, a hydatid cyst is seen in the pelvis with multiple internal daughter cysts (*white arrow*). **(B)** Non-contrast axial CT image confirming the presence of a hydatid cyst with multiple daughter cysts and showing areas of septal calcification (*white arrow*) adjacent to the iliac bone (*solid white arrow*). **(C)** Axial post-contrast CT revealing peripheral and septal enhancement (*white arrow*), which is characteristic of an active hydatid cyst classified as WHO type CE2. **(D)** Coronal short tau inversion recovery (STIR) MRI image of the pelvic cavity **(D)** demonstrating multiple hydatid cysts of varying sizes located within the pelvic cavity (*white arrows*) and the left paravertebral region (*white arrowhead*) of the lumbar spine. **(E, F)** Non-contrast **(E)** and contrast-enhanced **(F)** CT images of the neck showing an intraosseous hydatid cyst (*dashed white circle*) with internal septations involving the ramus and angle of the right mandible, resulting in medial displacement of the medial pterygoid muscle. Rim and septal enhancement is seen on the post-contrast image [*white arrow* in **(F)**].

**Figure 2:**
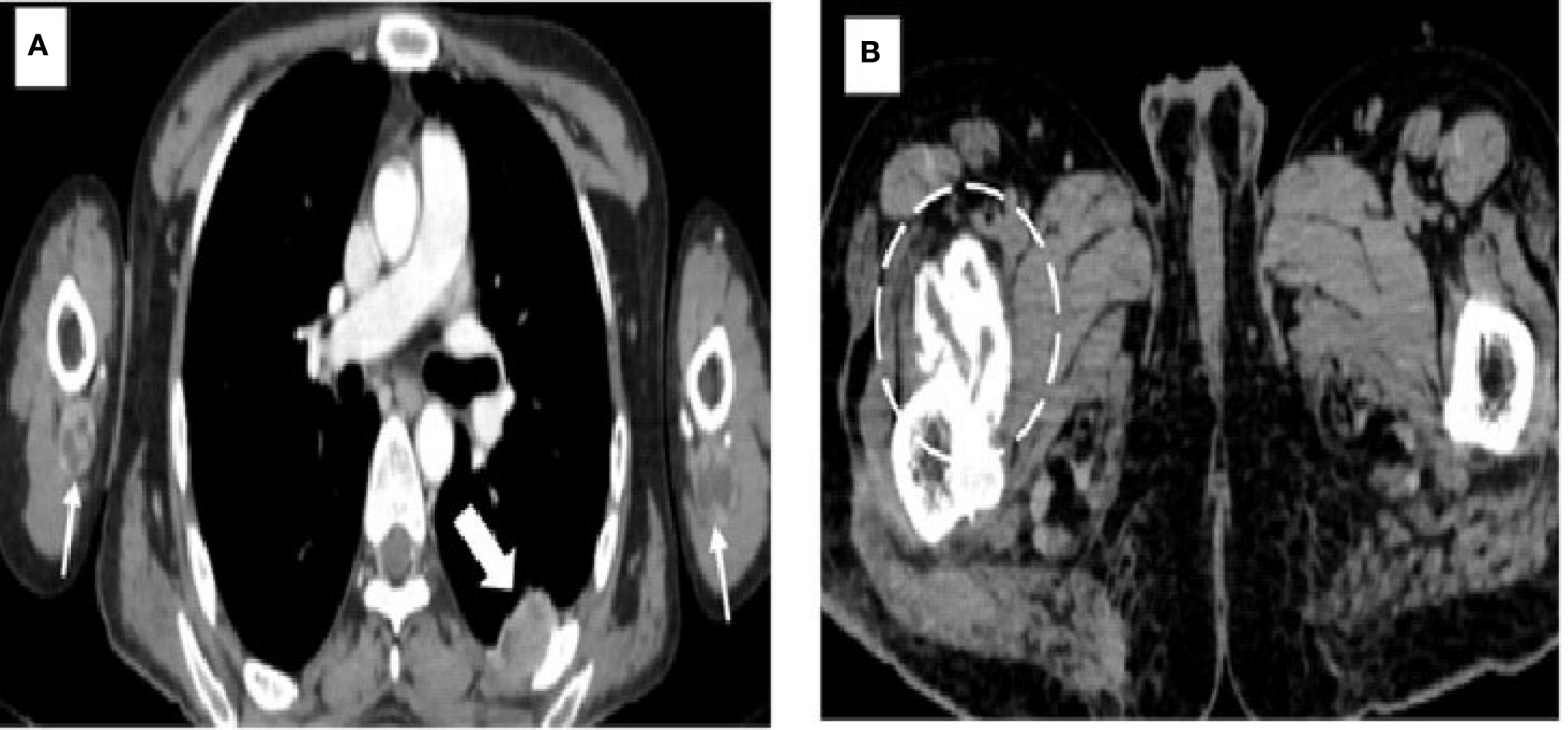
Images demonstrating hydatid cysts in the thorax, upper limbs, and pelvis. **(A)** Axial contrast-enhanced CT of the thorax showing a pleural hydatid cyst (*solid white arrow*) located posteriorly, with characteristic rim and septal enhancement. Additional hydatid cysts are seen in the extensor compartments of both arms (*white arrows*), situated beneath the triceps muscles. **(B)** Axial CT image of the pelvis revealing a well-circumscribed cystic lesion with calcified margins (*dashed circle*) and no internal daughter cysts, consistent with an inactive hydatid cyst (WHO type CE5). This lesion is located within the right iliopsoas muscle near its insertion at the lesser trochanter of the femur.

The patient initially underwent an ultrasound of the abdomen and other symptomatic regions, which revealed multiple (from four to five) well-defined, thick-walled cystic lesions in the pelvis (**Figure 1A**), the largest along the iliac bone measuring 5.3 cm × 6.5 cm × 7 cm [anteroposterior (AP) × transverse diameter (TD) × craniocaudal (CC)]. These cysts showed daughter cysts in a spoke-wheel pattern and hydatid sand, consistent with CE2 active-stage hydatid disease. Additional cystic lesions were identified in the suprasternal region, the anterior chest wall, the extensor compartments of both arms, and the right thigh. A contrast-enhanced computed tomography (CECT) confirmed these findings, demonstrating internal septations, daughter cysts, and septal calcifications in the pelvic cavity (**Figures 1B, C**), infraclavicular regions, pleura, and extremities (**Figures 2A, B**). Intraosseous cysts were also seen in the ramus and angle of the right mandible, measuring 3.4 cm × 4 cm × 3.4 cm (AP × TD × CC) (**Figures 1E, F**), showing a calcified rim without daughter cysts, corresponding to CE5 inactive cysts. CT additionally revealed multiple cysts at the craniovertebral junction, the cervical spine, and the dorsal spine, all showing peripheral rim enhancement, but no pericystic inflammation.

**Figure 3:**
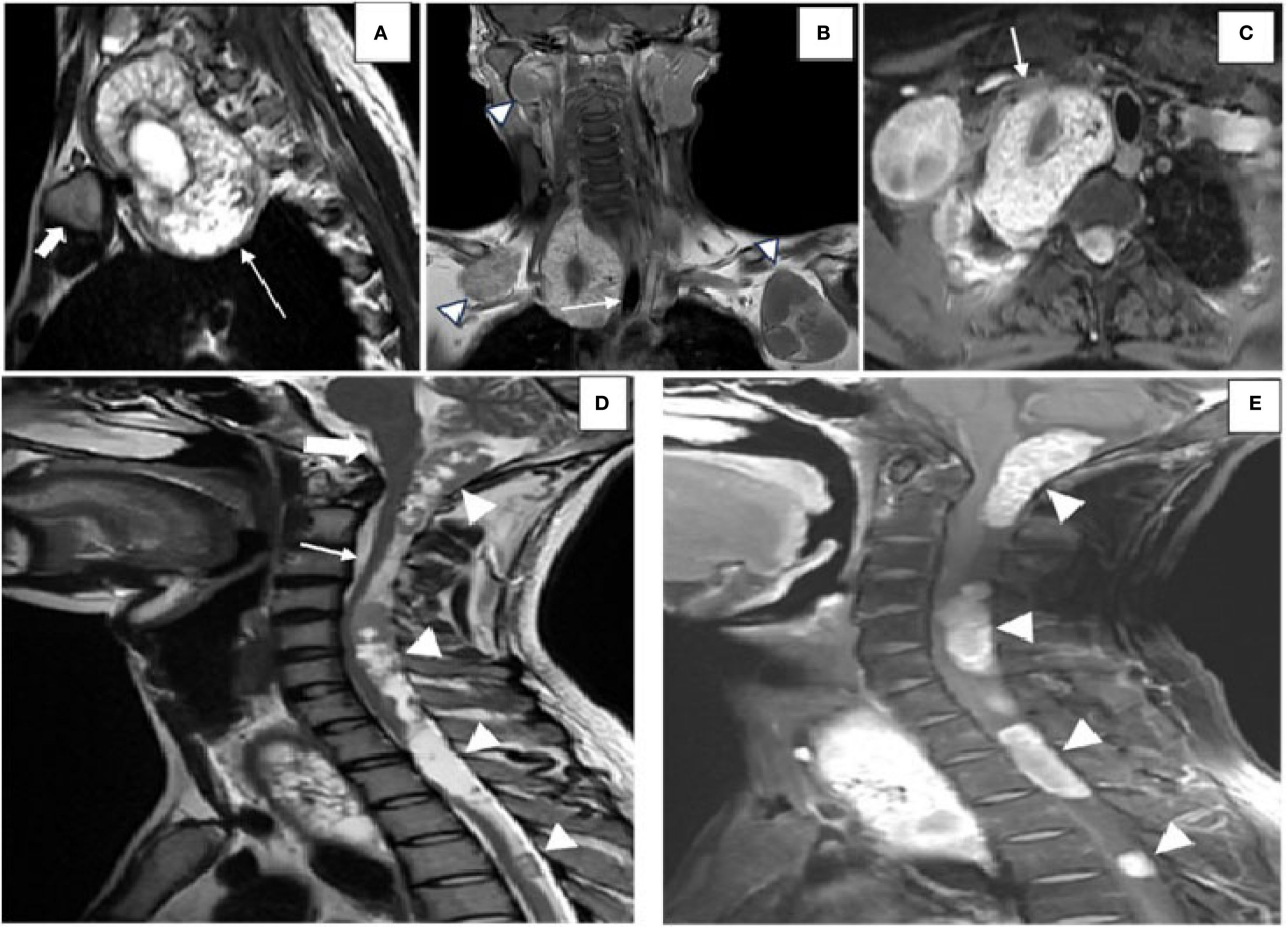
Images highlighting the MRI findings of hydatid disease involving the spine, mediastinum, and craniovertebral junction. **(A)** Sagittal T2-weighted MRI of the dorsal spine showing a hydatid cyst (*white arrow*) located in the suprasternal space, predominantly on the right side, positioned posterior to the medial end of the clavicle (*solid white arrow*) and extending into the superior mediastinum. **(B)** Coronal post-contrast T1-weighted image demonstrating the hydatid cyst displacing the trachea toward the left (*white arrow*). Additional hydatid cysts (*arrowheads*) are visualized in the anterior chest wall bilaterally below the clavicles, along with an intraosseous hydatid cyst involving the right mandible. **(C)** Axial post-contrast T1-weighted imaging revealing the hydatid cyst indenting the subclavian artery anteriorly (*white arrow*). [**(D)** Sagittal T2-weighted MRI image displaying multiple intradural, extramedullary hydatid cysts (*white arrowheads*)] located at the craniovertebral junction (*solid white arrow*), the cervical spine (*white arrow*), and the dorsal spine. These cysts are seen posterior to the brainstem and the spinal cord, exerting a mass effect by displacing the cerebellar vermis superiorly and compressing the spinal cord anteriorly. **(E)** Sagittal post-contrast T1-weighted image showing peripheral and septal enhancement of the hydatid cysts (*white arrowheads*), consistent with active disease.

MRI with gadolinium was performed to delineate the spinal and mediastinal extension. It revealed multiple intradural extramedullary T1-hypointense and T2-hyperintense cysts with daughter cysts involving the craniovertebral junction, measuring 1.7 cm × 2.3 cm × 5 cm (AP × TD × CC), and the cervical and dorsal spine, with the largest measuring 1.3 cm × 1.2 cm × 3.7 cm (AP × TD × CC) in the upper dorsal spine, showing post-contrast enhancement (**Figures 3D, E**), with clear compression of the cord. A large cyst was present in the superior mediastinum, measuring 5 cm × 5 cm × 8 cm (AP × TD × CC), and was seen displacing the trachea (**Figures 3A–C**). Some cysts demonstrated blooming on gradient recall echo (GRE) sequences, suggesting calcification. According to the WHO-IWGE classification, the spinal and mediastinal cysts corresponded to CE1 active unilocular cysts, while the chest wall and upper limb cysts with daughter cysts and calcification were CE2 active cysts. The right thigh cyst showed peripheral calcification without daughter cysts, consistent with CE5 inactive disease.

Serological testing was not performed due to financial constraints. However, a diagnosis of disseminated echinococcosis was made according to the WHO-IWGE imaging criteria, which permit imaging-based diagnosis in endemic areas when classic signs are present.

**Figure 4:**
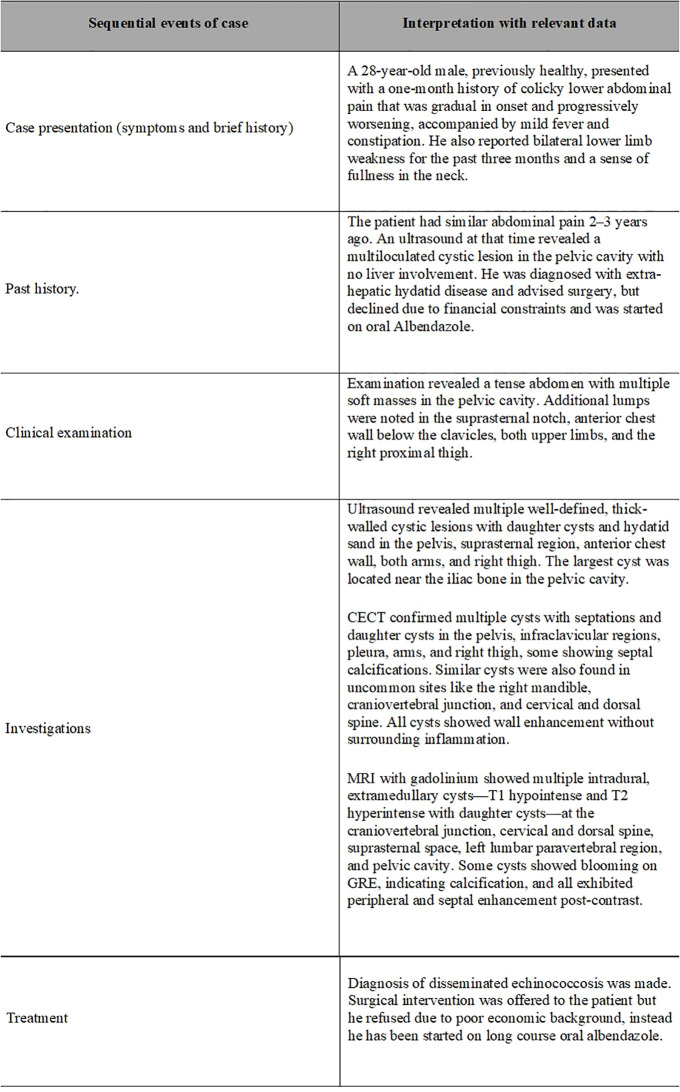
Table summarizing the sequence of events and timeline.

The patient was advised surgical excision of the spinal lesions, but he refused due to financial constraints. Puncture, aspiration, injection, re-aspiration (PAIR) was contraindicated due to the multiple septations, the location in the bone and epidural spaces, and the increased risk of spillage into the spinal canal. He was instead started on a long course of oral albendazole. The sequence of events is summarized in the **Figure 4**.

## Discussion

Hydatid cysts are benign, typically symptom-free fluid-filled lesions resulting from infection with the parasite *E. granulosus*. Dogs and sheep are the definite and intermediate hosts in the life cycle of *E. granulosus*, respectively. Humans are an accidental intermediate host (Karami et al., 2022). A hydatid cyst has three layers: the outermost layer, known as the pericyst, is formed by the host response to the infection and contains modified host cells, fibroblasts, giant cells, and eosinophils. Together, these cells form a rigid protective layer. The middle laminated membrane, known as the ectocyst, is an acellular structure. The inner most germinative layer, the endocyst, is thin and translucent, which produces daughter vesicles containing protoscolices (Mehta et al., 2016).

The time between infection and symptom onset can vary greatly. The clinical signs and imaging findings are often vague and are largely influenced by the organ involved, the size of the cyst, and its impact on nearby tissues. The liver is the most frequently affected organ, which is involved in 50%–70% of cases, followed by the lungs in 20%–30% (Ito et al., 2017). Other less commonly affected sites include the spleen (0.7%–8%), the kidneys (7%), the muscles (4%), the central nervous system (0.2%–3%), and the heart (0.2%–2%). Intraosseous involvement has been observed in <3% of cases (Maraimalai et al., 2023). Primary intradural extramedullary hydatid cyst of the spine is a rare form of the infection, causing neurological symptoms, and is commonly seen in developing countries (Lotfinia et al., 2013). The mediastinum is one of the rarest sites of hydatid disease, with incidence that varies from 0.5% to 2.5% (Msougar et al., 2013).

The diagnosis of hydatid disease can be made with a combination of history, examination, imaging findings, laboratory parameters, and histopathological findings. There is no serological test that is pathognomonic for echinococcosis. A negative serological test does not rule out hydatid disease, while a positive serology confirms a diagnosis only after visualization of a cyst with typical features of hydatid on US (Siles-Lucas et al., 2023). Therefore, the test result must always be compared with the imaging findings (Kaur et al., 2016).

US is considered as the best and most easily available, noninvasive imaging modality for the diagnosis of hydatid disease. US is used as a screening modality and for monitoring the effectiveness of treatment. An US scan can differentiate the hydatid sand, membranes, daughter cysts, and vesicles present inside the cyst. CT has more sensitivity (95%–97%) in demonstrating daughter cysts, the internal calcification, and the septa. It is also better for assessment of complications (Kaur et al., 2016). It also plays a role in imaging regions with a poor acoustic window for US or in patients with big body habitus or excess bowel gas shadowing. On MRI, hydatid cysts are hypointense on T1 and hyperintense on T2, but demonstrate a hypointense rim (due to the presence of a collagen-rich pericyst), which is the characteristic finding of hydatid disease. Diffusion-weighted imaging can help differentiate hydatid cysts from simple cysts as hydatid cysts show comparatively lower values on the apparent diffusion coefficient (ADC) map. MRI also helps in defining the extent of involvement of the surrounding soft tissue, particularly in cases of a ruptured hydatid cyst.

Several differential diagnoses were considered. Cysticercosis was excluded due to the absence of a scolex on MRI. Abscesses were excluded given the absence of fever, leukocytosis, rim enhancement, or pericystic inflammation. Simple cysts were unlikely given the presence of daughter cysts and hydatid sand. Tubercular cold abscesses, another regional mimic, were also ruled out by the lack of clinical evidence of tuberculosis and the absence of caseous necrosis or bony destruction typical for Pott’s spine. Necrotic metastases were unlikely given the patient’s young age, the absence of a primary malignancy, and the lack of irregular enhancing margins. Lymphangioma was ruled out as no fluid–fluid levels or non-enhancing septae were seen (Polat et al., 2003).

This case is notable for its extensive multi-organ involvement, far beyond the usual liver and lung presentation. Unusual sites such as the mediastinum (<4% of cases) and the bone, including the spine (0.5%–4%), made this combination particularly exceptional. The patient’s paraplegia from vertebral involvement highlights a rare but serious neurological complication of cystic echinococcosis. Finally, this case underscores the diagnostic value of correlating US, CT, and MRI, with each modality contributing complementary details that enriched the radiologic description of disseminated disease.

There is no single best treatment option for hydatid disease as all treatments are based on particular cyst characteristics and are stage-specific. There are three treatment options: 1) antiparasitic drugs such as benzimidazoles (albendazole and mebendazole), 2) PAIR), and 3) surgery.

CE1, CE2, and CE3 cases can be treated with medical therapy and/or PAIR. However, if the size is large, surgical intervention is preferred. PAIR is contraindicated in multiple organ involvement and for spinal, mediastinal, and cerebral cysts. CE4 and CE5 cysts are inactive stages and so are only kept under periodic observation.

The CDC/WHO recommend albendazole therapy in cases of disseminated hydatidosis or when surgical/PAIR options are infeasible (Centers for Disease Control and Prevention, 2024). The duration of albendazole treatment is decided based on the disease burden and the treatment response on follow-up imaging (Stojković et al., 2018). If the cyst is complicated, no matter the stage, surgery is the treatment of choice.

Previous reports of disseminated hydatid disease have described variable organ involvement and management approaches. Karami et al. (2022) reported on a case of intra-abdominal dissemination involving the liver, pelvis, and omentum, managed surgically with postoperative albendazole. Gupta et al. (2021) described a pediatric patient with liver, spleen, kidney, lung, and paraspinal muscle cysts who underwent surgical excision. Bennane et al. (2024) presented a case with hydatid cysts in the liver, spleen, kidneys, mediastinum, pancreas, and pelvis, treated with exploratory surgery and albendazole.

## Conclusion

Disseminated hydatid disease is a rare presentation of *Echinococcus* infection. However, when present, it can involve any part of the body where the blood reaches. Hydatid cysts in uncommon sites with unusual imaging findings can be difficult to diagnose. Familiarity with the imaging findings, especially in endemic countries, provides an important advantage in making a diagnosis (Polat et al., 2003). Hydatid cyst should always be kept as a differential diagnosis in mind when a cystic lesion is seen in an uncommon location with a clinical history indicative of hydatid disease.

No follow-up data are available to assess the efficacy of albendazole or the disease progression, which is a limitation of our case report. Future studies should track such cases to evaluate the long-term outcomes of medical therapy in disseminated hydatid disease.

## Data Availability

The original contributions presented in the study are included in the article/Supplementary Material. Further inquiries can be directed to the corresponding author.
